# A Critical Appraisal of Pyrolysis Pretreatment in Lithium‐Ion Battery Recycling: From High‐Temperature Material Transformations to Environmental Impact

**DOI:** 10.1002/cssc.70789

**Published:** 2026-06-07

**Authors:** Mohazzam Saeed, Lassi Klemettinen, Anna Klemettinen, Rodrigo Serna‐Guerrero

**Affiliations:** ^1^ Department of Chemical and Metallurgical Engineering School of Chemical Engineering Aalto University Espoo Finland

**Keywords:** green house gas emissions, lithium‐ion batteries, off‐gas, pyrolysis, recycling

## Abstract

Driven by sustainability goals and raw material scarcity issues, recycling of spent lithium‐ion batteries is becoming imperative. Pyrolysis is commonly employed for the removal of polymers to improve the liberation of active materials, yet its impact during the thermal degradation remains unexplored. Thus, the present study investigates the effect of pyrolysis time and temperature on the liberation and composition of “black mass (BM).” Thermal decomposition of various size fractions of an industrial BM was studied using thermogravimetric analysis and bench‐scale pyrolysis, while characterization before and after pyrolysis was performed using X‐ray diffraction and scanning electron microscopy. Furthermore, analysis of gaseous byproducts during pyrolysis using Fourier transform infrared offered insights on the decomposition byproducts and their environmental impact. It is hereby demonstrated that, while pyrolysis results in the partial degradation of polymeric binders, this is not the result of pure thermal degradation. Indeed, graphite, polymers, and Al reduce cathode active materials into metallic species via in situ carbothermic or aluminothermic reduction, producing Al_2_O_3_, CO_*x*_, and alkanes. The experimental results are rationalized into a reaction mechanism for pyrolysis of black mass, revealing that pretreatment via pyrolysis can compromise the preservation of battery active material and needs to be carefully considered during direct recycling routes.

## Graphical abstract

Pyrolysis of different size fractions of waste lithium‐ion batteries reveals complex interaction among cathode materials, graphite, aluminum, and polymeric binders. Carbothermic and aluminothermic reduction pathways promote the formation of metallic species at moderate temperatures. Characterization of pyrolysis products further identifies gaseous emissions, including greenhouse gases, providing insight into the mechanisms governing battery thermal decomposition under inert atmosphere.

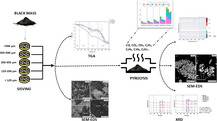

## 1 Introduction

With the increasing demand of raw materials for the manufacturing of lithium‐ion batteries (LIBs), it is necessary to develop efficient recycling methods to alleviate the depletion of natural resources and to treat the large material volumes expected to reach their end‐of‐life in the coming years. Pyrometallurgy and hydrometallurgy routes have been widely explored to recycle spent LIBs [1, 2, 3, 4]. Pyrometallurgy operates at elevated temperatures and offers process flexibility, faster chemical reactions, and scalability is considered a well‐established technology. However, it brings several challenges, such as intensive energy consumption, hazardous gas emissions, the degradation of graphite, and the loss of Li and Mn into slag [2, 5, 6, 7]. In contrast, hydrometallurgical processes (e.g., leaching, solvent extraction, and precipitation) utilize low temperatures and can provide high metal recovery rates for metals such as Li, Co, Ni, and Mn. Yet, they bring several concerns including intensive water usage, hazardous chemical consumption, and the need for pretreatment stages [8, 9, 10]. Recently, froth flotation has gained attention as a direct recycling method under the premise that anode and cathode materials can be separated based on their differences in surface wettability [11, 12, 13]. Through froth flotation, recycling of both cathode and anode active materials is possible without modification of their chemical composition. However, this route is still in the development phase with challenges including sensitivity to feed material composition, grain size, and surface chemistry, the need for binder removal, and higher water consumption [14, 15].

The liberation of active materials through binder removal is imperative for the efficient separation of species through hydrometallurgical and direct recycling routes [16, 17]. The presence of binder in the leaching and flotation wastewater is suspected to cause serious health problems [18, 19], whereas in the case of pyrometallurgy, it can produce hazardous gases such as HF and loss of calorific value [20, 21]. Owing to the strong chemical resistance of the binder material, its removal requires dedicated pretreatment stages [21]. Various methods have been reported for binder removal including the use of organic solvents [22, 23, 24], alkaline leaching [25], cryogenic grinding [26, 27], ultrasonic treatment [28, 29], application of high shear forces [30], and Fenton oxidation [31]. However, these approaches are limited, either by low efficiency or environmental and safety concerns.

Thermal pretreatment processes, particularly pyrolysis, stand out as a prominent method for binder removal. According to some studies, thermal pretreatment is efficient and offers simple operation compared to other alternatives [18, 32]. It has also been experimentally demonstrated that employing pyrolysis leads to enhanced flotation efficiency [18, 33, 34]. So far, key parameters such as temperature and time have been explored to refine the pyrolysis process, but contradictions still exist regarding the effectiveness of these conditions, particularly in the context of metals recovery and binder decomposition. For instance, Xiao et al. [35] illustrated that at 400°C, the C—F bonds start to break down along with the conversion of graphite to CO_2_/CO, resulting in partial decomposition of polyvinylidene fluoride (PVDF). Zhang et al. [18] reported that 500°C and 10 min are sufficient to thoroughly decompose binder and effectively liberate the electrode materials. Further work by Zhang et al. [34] indicated that, while the binder decomposes at 500°C, residual binder oil persists on the surface of LiCoO_2_, only fully decomposing at 550°C. Wang et al. [21] found that binder decomposition occurs at 475°C, though no specific pyrolysis time was provided. Similarly, Li et al. [36] found that binder decomposition occurs within the 260°C–600°C temperature range. Zhu & Chen [37], reported a two‐stage pyrolysis pretreatment process, where decomposition of organic solvents occurred at 150°C while binder was removed at 450°C. The pyrolysis time for both stages was kept at 30 min. In contrast, Lombardo et al. [16] stated that pyrolysis of black mass (BM) at 600°C–700°C completely removes the binder following carbothermic reaction. Siame et al. [38] examined froth flotation behavior of pyrolyzed BM and showed that, by employing 10 wt% of CaO at 400°C, liberation of cathode material was improved along with graphite selectivity during froth flotation. Furthermore, Huang et al. [33] reported that after 30 min of pyrolysis at 400°C–500°C, the binder does not decompose, but instead forms a liquid film that covers the surface of electrode materials, inhibiting further decomposition. Pražanová et al. [39] investigated pyrolysis pretreatment for NMC 622 across 400°C–650°C and demonstrated that 500°C offers the most effective trade‐off between impurity removal and cathode preservation. These conflicting findings highlight the need for comprehensive study to better understand how various parameters impact the pyrolysis pretreatment of spent LIBs.

To further complicate the analysis of thermal treatment of LIBs, recent studies report variations in the composition of BM within different size fractions [40, 41, 42, 43]. Recently, Tas et al. [44] evaluated recycling routes of spent LIBs underscoring the need to consider variable feed composition, feed size, and reaction pathways along with the off‐gas treatment systems in addressing the environmental impacts of pyrolysis. Thus, understanding the behavior of BM with varying composition in different size fractions is imperative to identify the right pyrolysis parameters. This will help develop strategies for the efficient recycling of spent LIBs using size‐dependent processes such as froth flotation, leaching, or solvent extraction. A comprehensive investigation into the off‐gas emission is thus essential, as hazardous emissions during pyrolysis could be a substantial risk to the worker safety along with negative environmental impacts.

The present work aims to provide a comprehensive analysis on the effects of pyrolysis as a pretreatment stage for LIB recycling by: i) identifying the underlying chemical reactions and degradation pathways during pyrolysis and the extent of binder removal; ii) offering insights into the size‐dependent behavior of BM; and iii) characterizing the gas emissions and their environmental impact. The obtained information can thus play key role for the development of off‐gas control system such as scrubbers, thermal oxidizers, and activated carbon, etc. for the spent LIBs recycling processes.

## 2 Materials and Methods

Spent BM was obtained from Akkuser Oy (Nivala, Finland). According to the publicly available documentation of this pretreatment process [45], no discharging or preseparation of electrodes were done and the collected LIBs underwent size reduction via two‐stage crushing. During the first crushing stage, the temperature was kept at 40°C–50°C to avoid potential risks of fire or explosion. The obtained material was then subjected to magnetic separation to remove iron sheets, followed by sieving to classify material into overflow and underflow fractions. The fine size underflow product (<1.25 mm) was the material used in the present study.

### 2.1 Sieving and Characterization

The BM was fractioned using a vibratory sieve shaker (Fritsch, Analysette 3, Idar‐Oberstein, Germany). The particle size fractions were obtained by dry mechanical sieving using a stacked set of standard test sieves with nominal apertures of >1000, 500–1000, 350–500, 250–350, and <125 µm, following the procedures specified in ASTM E11 for laboratory sieving. Following previously reported methods [46], sieving was performed using a batch size of 200 g under an amplitude of 6 mm for 15 min to obtain optimal particle separation and minimal clogging. After sieving, five different size fractions were obtained: >500, 355–500, 250−355, 125–250, and <125 µm. The particle size distribution curve of raw BM feed is attached in Figure S1.

The elemental compositions of the BM fractions were analyzed using ICP‐MS commissioned to an external laboratory (X‐ray mineral services, UK). Samples were digested using CEM Mars 6 microwave with iPrep vessels, using an in‐house modified version of the ‘clay’ method supplied with the system. The acid mixture used was dominated by high‐purity HNO_3_ with subordinate HCl. Elemental concentrations were analyzed with iCAP TQ inductively coupled plasma mass spectrometry (ICP‐MS) using a mixture of no gas, He (KED) and O2 reaction modes optimized to best suit each analyte in an in‐house developed method. Matched, synthetic calibration standards were used in quantification. Table 1 presents the compositional results for the various BM size fractions.

**TABLE 1:** Mass fraction (%) and elemental compositions (wt%) of principal metals within size fractions.

Sieve size	Mass, %	Li	Co	Ni	Cu	Al	Fe	Mn	C
>500	22.14	2.66	16.15	1.72	9.69	7.75	0.96	1.63	14.10
355–500	7.15	3.67	22.58	2.15	5.22	3.25	0.40	2.32	11.20
250–355	9.15	4.11	25.97	2.44	3.35	1.86	0.45	2.55	16.60
125–250	19.7	4.09	25.50	2.26	1.48	1.08	0.38	2.26	18.50
<125	41.5	3.61	22.18	2.03	0.48	0.86	0.33	2.18	17.90

The X‐ray diffraction (XRD) analyses were also commissioned to an external laboratory (X‐ray mineral services, UK). The samples were hand crushed to a fine powder and pressed into a steel sample holder for presentation to the X‐ray beam. Each sample was analyzed before and after being spiked with a silicon crystalline powder, used as internal standard, for the determination of the amorphous content. The characterization was conducted with a Malvern Panalytical X’Pert3 diffractometer from 4.5 to 75 2θ using Cu K radiation at 40 kV and 40 mA. The samples were analyzed for 2 h at a step size of 0.013°. Phase identification and quantification, using the Rietveld method, were carried out using HighScore Plus (v.4.9 by Malvern Panalytical) equipped with ICSD database and PDF‐4 Minerals 2024 database.

Thermogravimetric analysis (TGA) was conducted using a Netzsch STA 449 apparatus. Representative samples of each size fraction, weighing 25 ± 5 mg, were loaded individually on an alumina crucible. A high‐purity He atmosphere (≥99.99%) was maintained with a flow rate of 70 mL/min for an inert environment. Temperature was increased from ambient to 1000°C at a rate of 10°C/min.

Scanning electron microscopy with backscattered electron (SEM‐BSE) analyses were performed with Mira 3 SEM (Tescan, Czech Republic). Samples were imaged in backscattered electron mode as powders on carbon tape and as polished cross‐sections after casting the powders in epoxy resin, grinding, polishing, and carbon coating. The SEM acceleration voltage was set to 15 kV, and beam current to 2 nA (for powders on carbon tape) or 5 nA (for polished cross‐sections in epoxy). Selected areas of the powders and polished cross‐sections were analyzed using energy‐dispersive spectrometry (EDS, Oxford Instruments, UK). Before EDS analysis, a beam measurement was conducted using pure Cu.

The presence of PVDF functional groups was analyzed using PerkinElmer Fourier transformed infrared spectroscopy (FTIR) integrated with attenuated total reflectance (ATR). Measurements were performed in transmittance mode with the wavenumber fixed at 500–4000 cm^−1^, resolution of 4 cm^−1^ and 16 scans. Both background calculations and scans were performed under similar conditions.

### 2.2 Pyrolysis

**FIGURE 1:**
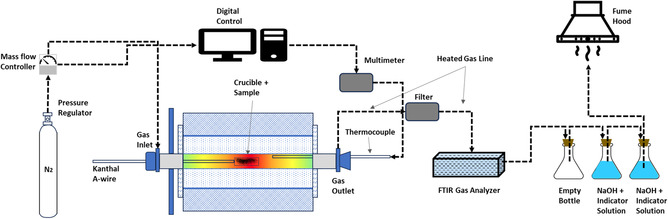
Schematic of pyrolysis experimental setup.

Figure 1 shows a schematic representation of the experimental setup used for the pyrolysis experiments, featuring a horizontal LTF12/50/610 tube furnace (Lenton, UK). The furnace had an alumina work tube with a length of 820 mm and inner diameter of 38 mm and was equipped with FeCrAl heating elements. During the experiments, a constant flow of 200 ml/min of N_2_ gas (99.999% purity) was used to maintain an inert, oxygen‐free atmosphere inside the furnace. The gas flow rate was regulated with a digital mass flow controller (DFC26, Aalborg, USA). Temperature was measured continuously with an S‐type thermocouple (Johnson‐Matthey Noble Metals, UK), positioned in the hot‐zone of the furnace, and connected to a Keithley 2000 multimeter (Keithley, USA). Cold junction compensation was performed using a Pt100‐sensor connected to Keithley 2010 multimeter (Keithley, USA). Both ends of the furnace work tube were cooled with water while the outgoing gases were directed to a gas analyzer, after which the gases were bubbled through an aqueous solution of NaOH in trap bottles to absorb some of the possible harmful gases.

The furnace was preheated to the target temperature and then a silica boat (inner length 120 mm, inner width 25 mm, height 6 mm) was placed in the cold zone of the furnace. At each measurement, the crucible held 5 g of BM sample. The furnace was then sealed and purged with nitrogen for 10 min to remove any residual air inside the furnace. After 10 min, the boat was slowly pushed towards the hot zone of the furnace in three steps (2 × 2‐minute breaks in between) to avoid crucible cracking due to thermal shock and to eliminate thermal lag [47]. After reaching the hot zone, the sample was kept there for 15, 30, 45, or 60 min. The removal of the sample from the hot zone followed the aforementioned procedure in reverse order. After pyrolysis, the sample was weighed again to determine mass loss.

Throughout the pyrolysis treatment, gas emissions were continuously monitored using a gas analyzer (DX4000 FTIR, Gasmet, Finland) connected to the off‐gas pipeline of the furnace. Multiple species, including CO, CO_2_, HF, CH_4_, C_2_H_4_, C_2_H_6_, C_3_H_8_, and C_6_H_14_ were monitored. With Calcmet software, real‐time concentrations of these off‐gases were quantified. In a single measurement cycle, the off‐gas was initially pumped into the gas analyzer for 30 s, followed by a measurement period of 20 s. 1400 ml/min N_2_ was used as dilution gas to increase the total gas flow to the analyzer.

The experimental error was estimated using the emission values obtained from three replicate runs and combining the standard deviation of the measurements with the nominal instrument error according to the manufacturer's specification (i.e., 3%) using a root‐sum‐of‐squares approach:

(1) $$\text{Total uncertainity}=\sqrt{{\left(\frac{\mathrm{SD}}{\sqrt{n}}\right)}^{2}+{\left(0.03\times \text{mean}\right)}^{2}}$$

where SD is the standard deviation, *n* is the number of experimental runs, and mean is the arithmetic mean value of emissions obtained from all runs.

## 3 Results and Discussion

### 3.1 Thermogravimetric Analysis

**FIGURE 2:**
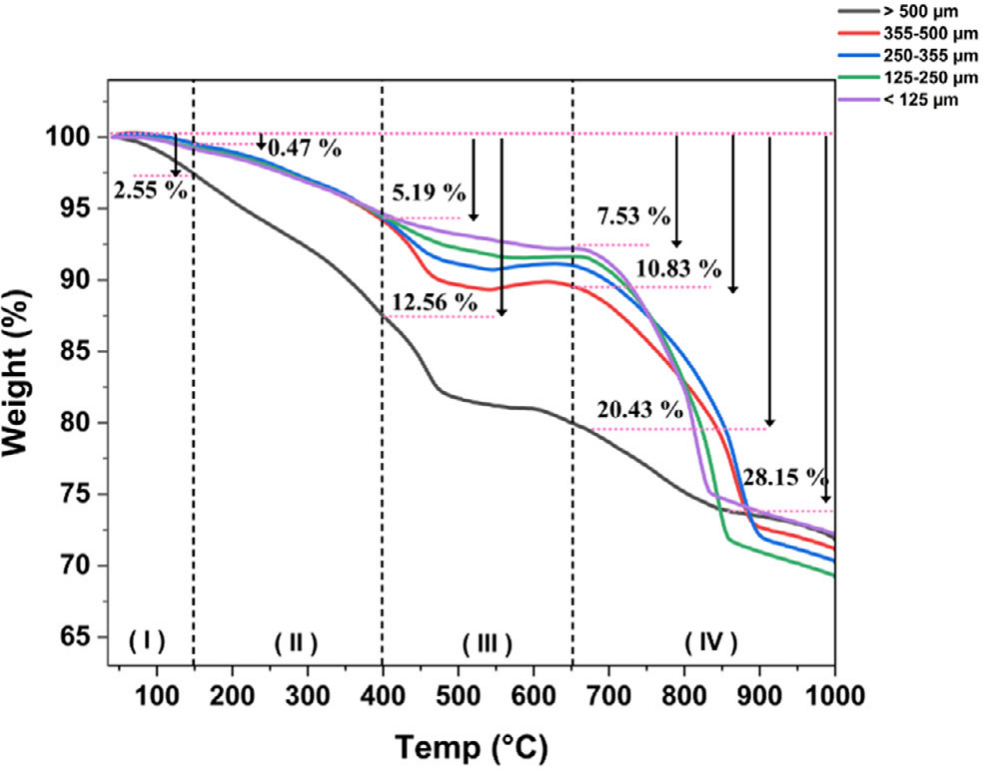
TGA analyses of various size fractions of spent LIBs components.

TGA was conducted to accurately determine the mass loss across various size fractions throughout a wide range of temperatures. As depicted in Figure 2, between room temperature and 1000°C, the thermal degradation profile can be divided into four distinctive stages of thermal degradation.

The mass loss observed at each stage can be attributed to the temperature‐dependent decomposition of specific battery components. The first stage, within a temperature range of 40°C–150°C, primarily involves the loss of moisture and partial degradation of electrolytes. The second stage of thermal degradation (i.e., 150°C–400°C) likely corresponds to the decomposition of carbonate‐based electrolytes into propylene carbonate (310°C), dimethylene carbonate (180°C–300°C), and diethylene carbonate (330°C) [48]. Additionally, partial decomposition of separators such as PP (270°C–470°C) and PE (430°C–480°C) is plausible [49]. A third stage of thermal degradation is observed between 400°C and 650°C, representing the decomposition of binder material (i.e., PVDF), as well as the chemical reduction of metal oxides via carbothermic reaction with graphite. Therefore, the weight‐loss profiles observed in this temperature range are of particular interest due to their relevance in the liberation of active materials. Stage four, 650°C–1000°C, is characterized by several thermal transformations, which may be undesired, including melting of Al, graphite oxidation, and the decomposition of LiCO_2_ into Li_2_O and CO_2_ [50, 51].

While all size fractions consistently exhibited mass losses, their extent at each stage of thermal degradation shows notable differences (Figure 2). During the first two stages, the >500 µm size fraction exhibited a combined weight loss of 12.6% in contrast to 5.2% in finer fractions. This indicates that moisture, electrolytes, and separators are present at higher concentrations in the larger particles. In stage III, the finer fractions (i.e., <125, 125–250 and 250–355 µm) exhibit lower weight loss percentages compared to larger particles (355–500 and >500). This suggests that electrolyte, separators, and plastic residue strongly influence weight loss. These observations are further corroborated by the SEM analysis, as will be discussed in Section 3.3. In stage IV, the finer fractions (<355 µm) show higher weight loss compared to the coarser ones. This likely reflects the varied distribution of carbon‐containing components across various size fractions. The carbon content in smaller size fractions predominantly belongs to graphite, whereas larger size fractions also contain electrolytes, separators, and plastics as carbon containing species. As will be further detailed in Section 3.3, the evolution of CO and CO_2_ gases detected in Stage III implies the degradation of various organic substances but also an undesired carbothermic oxidation of graphitic carbon.

### 3.2 Mass Loss during Pyrolysis

**FIGURE 3:**
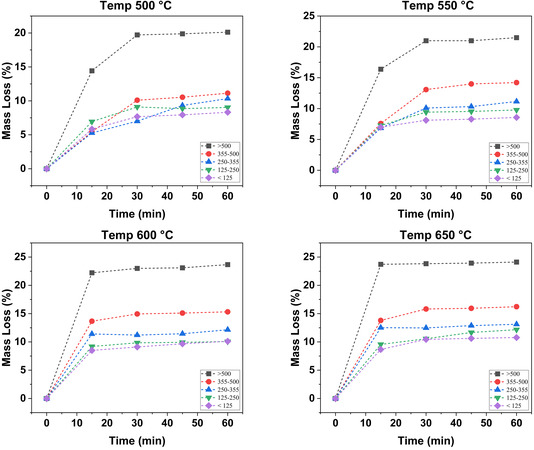
Mass losses of the different size fractions at different pyrolysis temperatures as a function of time.

The mass loss of the different BM size fractions (>500, 355–500, 250−355, 125–250, <125 µm) under various pyrolysis temperatures (500°C, 550°C, 600°C and 650°C) and reaction times (15, 30, 45, and 60 min) is shown in Figure 3. In a similar fashion to the TGA observations discussed in Section 3.1, the mass loss rate varied among fractions, thus corroborating a size‐based response to temperature changes. Indeed, the larger size fractions consistently presented higher mass losses under all conditions of pyrolysis time and temperature explored in the present study.

The results in Figure 3 show that mass loss is promoted at higher pyrolysis temperatures, an observation that is in line both with the TGA data shown in Section 3.1 and with other reports in the scientific literature (Lombardo et al. 2019). For example, at 500°C and 15 min, the coarsest size fraction presented a weight loss of 14.4%, which increased to 23.7% at pyrolysis temperature of 650°C and 15 min of pyrolysis. While such increase in mass loss is less dramatic for smaller size fractions, it is invariably observed. For instance, the finest fraction (<125 µm) displayed a weight loss of 5.8% at 500°C, increasing to 10.2% at pyrolysis temperature of 650°C after 15 min of pyrolysis time.

Figure 3 also demonstrates that the degradation kinetics are temperature dependent, i.e., higher temperature leads to faster degradation while it is gradual at lower temperatures. For all size fractions, the maximum mass loss at 500°C was obtained after 30 min while it only takes 15 min at 650°C to reach a plateau. Given that the mass loss change at all temperatures was negligible after 30 min of pyrolysis, the subsequent analyses (SEM, FTIR, XRD, and environmental impact) were conducted on samples pyrolyzed for this time length.

### 3.3 Microstructural Analysis

#### 3.3.1 Industrial Black Mass

**FIGURE 4:**
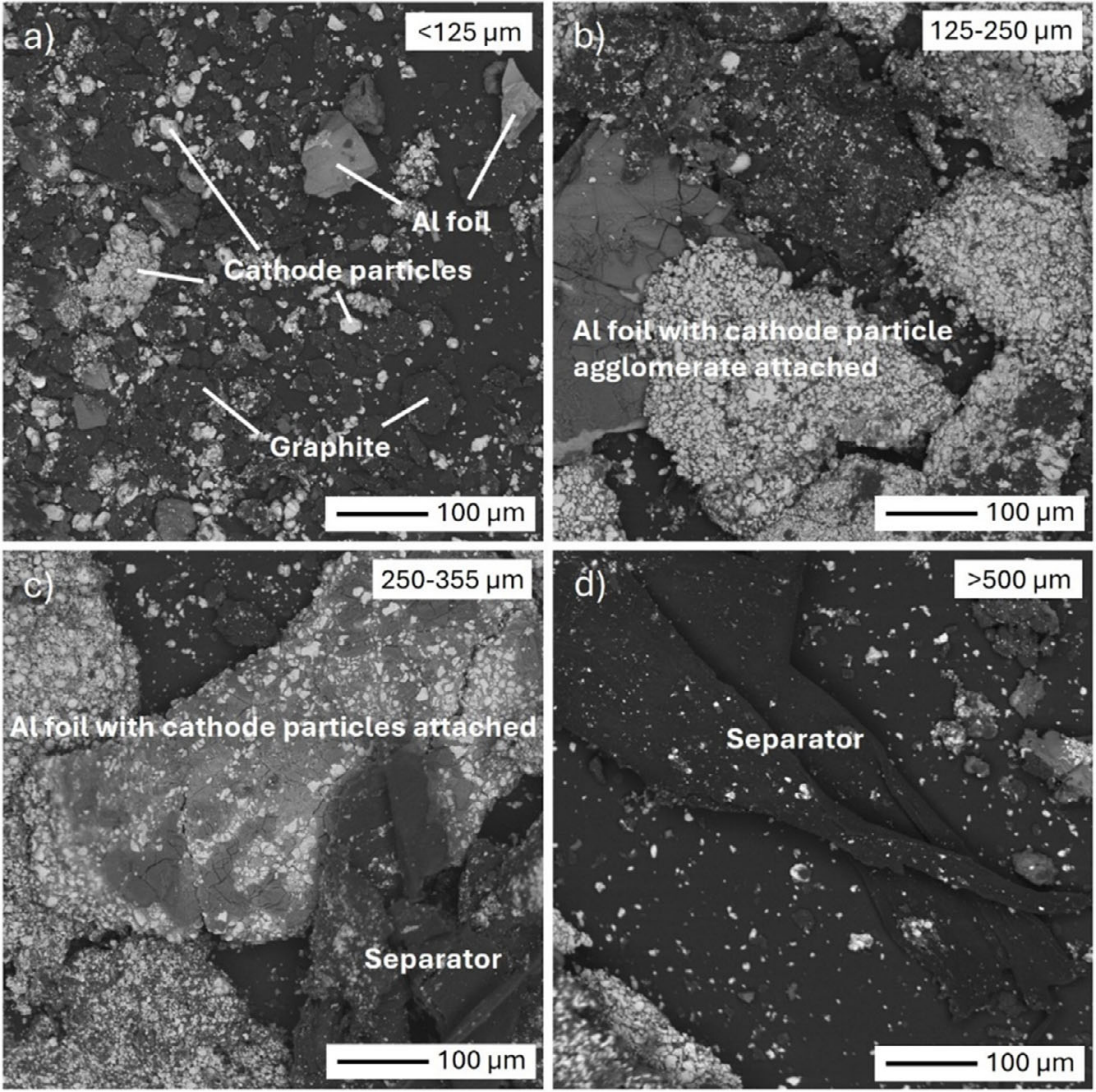
SEM‐BSE images of BM fractions before pyrolysis: (a) <125 μm, (b) 125–250 µm, (c) 250–355 μm, and (d) >500 µm.

Representative images obtained using SEM‐BSE of the various BM size fractions before pyrolysis are shown in Figure 4, and EDS spectra of different particles are presented in Figure S2. These BSE images were taken from powders dispersed on top of carbon tape, as it was of interest to identify any difference in particle morphologies at each size fraction. As seen, in the smallest size fraction (<125 μm), black spheroidized graphite particles are visible and can be easily differentiated from light‐gray particles of active cathode material. The presence of fine Al foil particles was also identified. Spheroidized graphite particles were comparatively more abundant in the smallest size fraction. These graphite particles appear well liberated, although ultra‐fine cathode crystals were often found on top of them. Across all size fractions, the cathode material is present both as individual particles as well as aggregates. In Figure 4, a notable presence of cathode material aggregates is visible when the particle size of the fraction increases. In the largest size fractions (Figure 4c,d), carbon‐rich separator sheets of irregular shapes were also present. Although a similar carbon content was measured in all size fractions (Table 1), the SEM images clearly suggest that they correspond to different species in coarse and fine particles. Indeed, graphitic carbon preferentially reported to the smallest size fractions, while polymeric carbon was retained in the larger size fractions. A partial explanation can be associated with their differences in mechanical properties. Zhang et al. [52] reported that anodic graphite has lower failure strain (0.08) than polymeric separator (0.731). As graphite is more brittle, it breaks into smaller particles while polymeric separators which are transformed into larger flakes. Thus, after grinding, the difference in the mechanical behavior of the carbon species in battery material leads to variation in their size distribution.

#### 3.3.2 Pyrolysis Products

**FIGURE 5:**
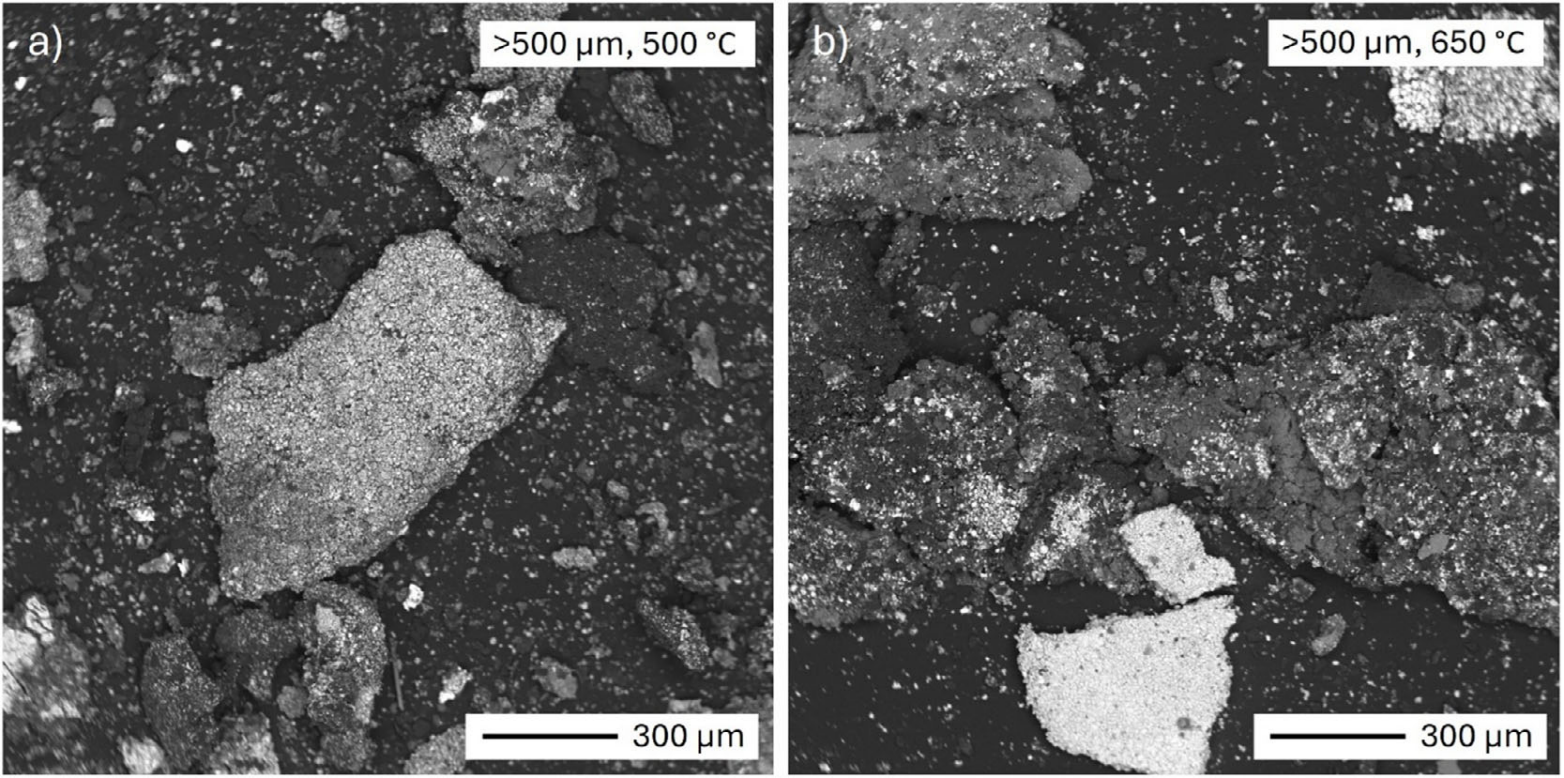
SEM‐BSE images of >500 µm powder samples after pyrolysis at 500°C and 650°C for 30 min. The number of small particles (liberated graphite and cathode material particles) has somewhat increased, and the separators have been removed.

SEM‐BSE images of the BM fraction with particle size >500 µm after pyrolysis at 500 and 650°C are shown in Figure 5. A noticeable observation is the disappearance of large, irregularly shaped dark foils, previously identified as plastic separators (Figure 4). This is well in agreement with the TGA analysis (Section 3.1), suggesting that separator is removed during pyrolysis, consistent with findings in the literature [53]. After pyrolysis, the presence of individual carbon (graphite) particles was also detected, suggesting the liberation of fine, individual graphite particles from larger aggregates. Since binder holds the aggregates together, the liberation of particles could be indirectly associated with binder decomposition. As will be discussed in Section 3.5, this is consistent with FTIR results, which confirm thermal breakdown of binder at 650°C. Since some cathode material aggregates were still present after pyrolysis, it cannot be stated from SEM‐EDS analysis that binder has been completely removed, even after pyrolysis at 650°C. More detailed characterization regarding the binder is presented in Section 3.5.

**FIGURE 6:**
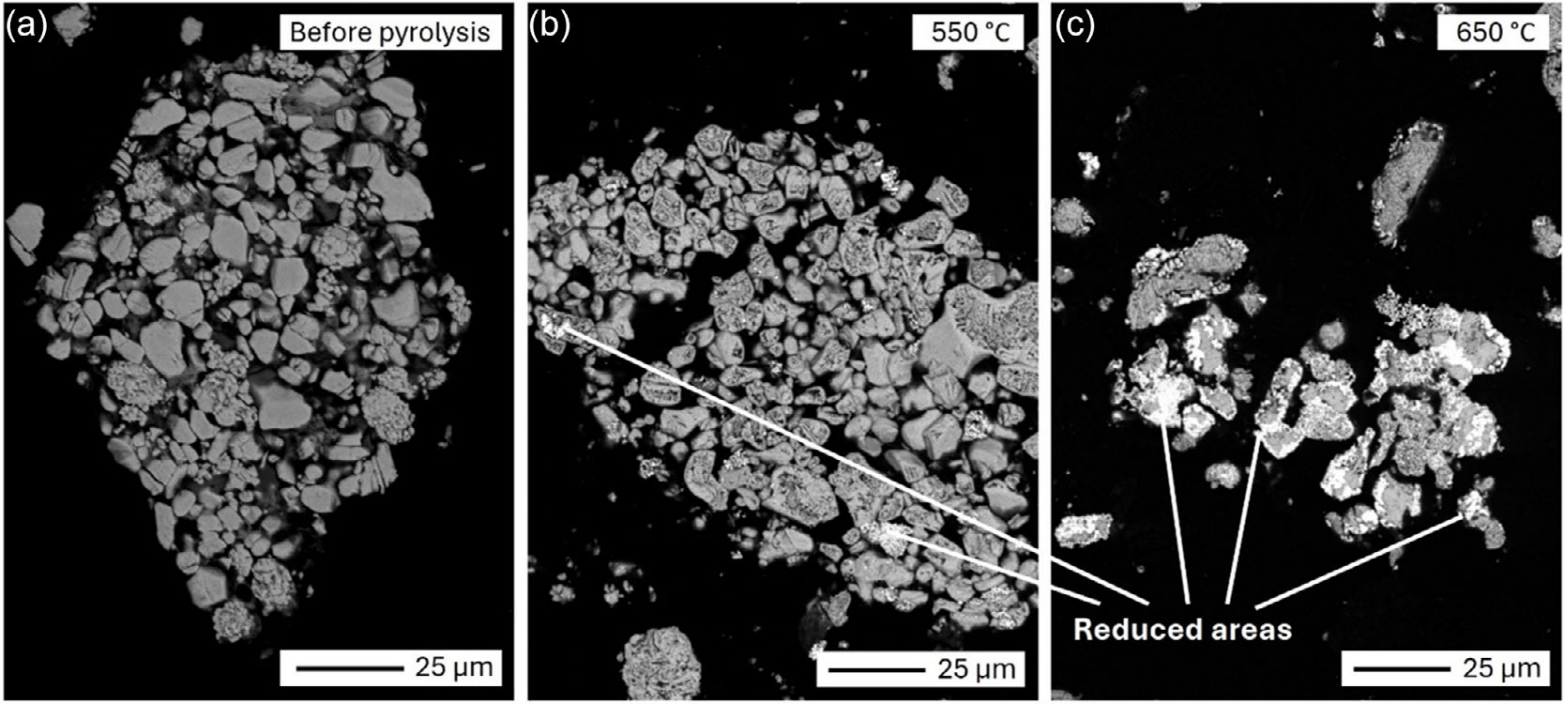
SEM‐BSE images of cathode particle aggregates in polished cross‐sections: (a) <125 μm BM fraction before pyrolysis, (b) after pyrolysis at 550°C, and (c) after pyrolysis at 650°C. The images clearly show that cathode particles undergo increased reduction as the pyrolysis temperature is increased.

Regarding the behavior of finer particles, selected images of cathode particle aggregates in the <125 μm size fraction are shown in Figure 6, before and after pyrolysis at 550°C and 650°C. Unlike Figures 4 and 5, these images are taken from polished cross‐sections, revealing both the surfaces and bulk composition of the cathode particles. A noteworthy observation is the increased brightness of cathode particles at higher temperatures, reflecting a higher average atomic number. EDS analysis (Figures S3 and S4 as well as Table S1) revealed that these brighter areas consist of metallic Co, indicating partial reduction of the cathode metal oxides. The reduced areas were mostly seen on the edges of the particles, suggesting that the reduction started from the surface and progressed towards the center of the particles. Since PVDF binder resides on the surface, its proposed decomposition, along with the reduction of LiCoO_2_ to CoO (described later in Section 3.5) support these observations. Since pyrolysis was performed in an inert environment, it is only reasonable to conclude that the metal oxide reduction results from their reaction with carbon‐containing species. Carbothermic reduction during pyrolysis has also been reported in previous literature [54, 55]. Park et al. [56] calculated Gibbs free energies of various reactions between LiCoO_2_ and carbon and noticed that these reactions become thermodynamically favorable at temperatures above 600°C, supporting the hypothesis about carbothermic reduction of cathode particles at high pyrolysis temperatures.

Another observation from Figure 6 is that the individual cathode particles within the aggregates seem to be better liberated after pyrolysis at 650°C, compared to pyrolysis at 550°C and especially to the sample before pyrolysis. Better liberation indicates the removal or decomposition of binders at least to some extent. Even though Figure 6a–c does not show the exact same aggregate, these are representative images reflecting a behavior observed throughout the samples after pyrolysis, especially at 650°C.

**FIGURE 7:**
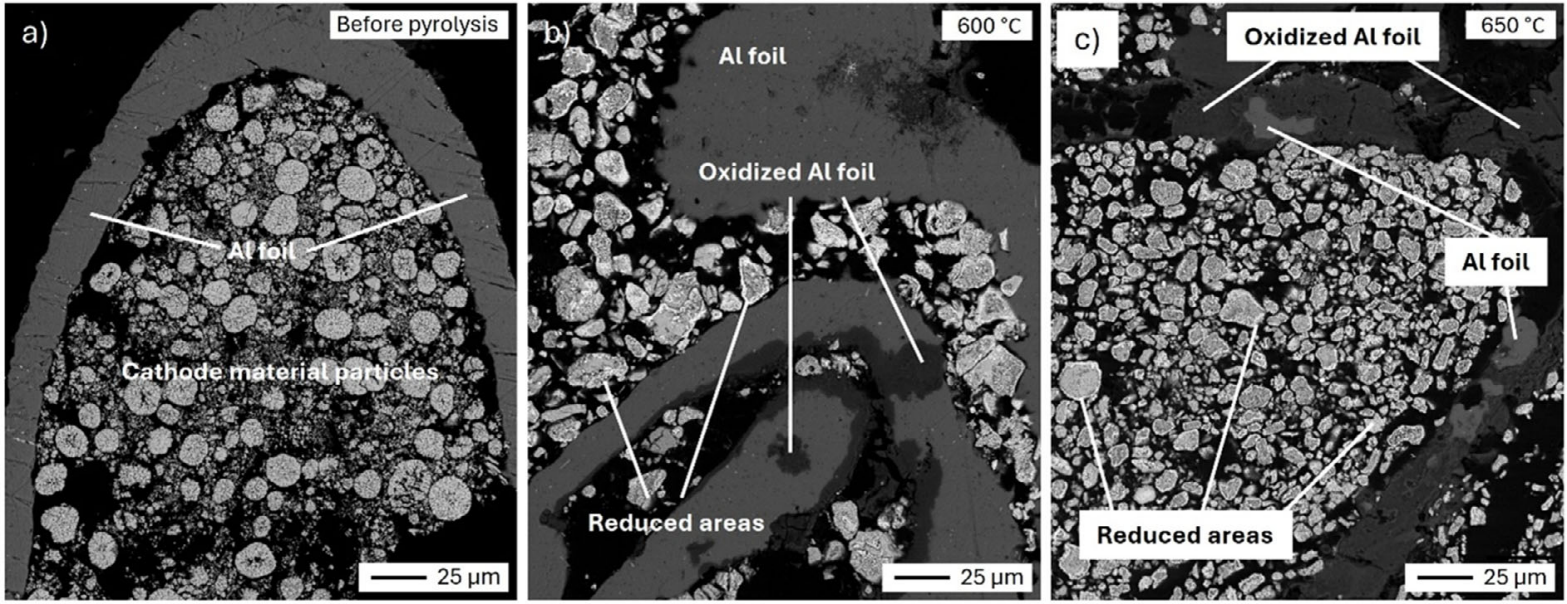
SEM‐BSE images of selected regions of >500 μm size fraction in polished cross‐sections: (a) before pyrolysis; (b) after pyrolysis at 600°C; and (c) after pyrolysis at 650°C.

**FIGURE 8:**
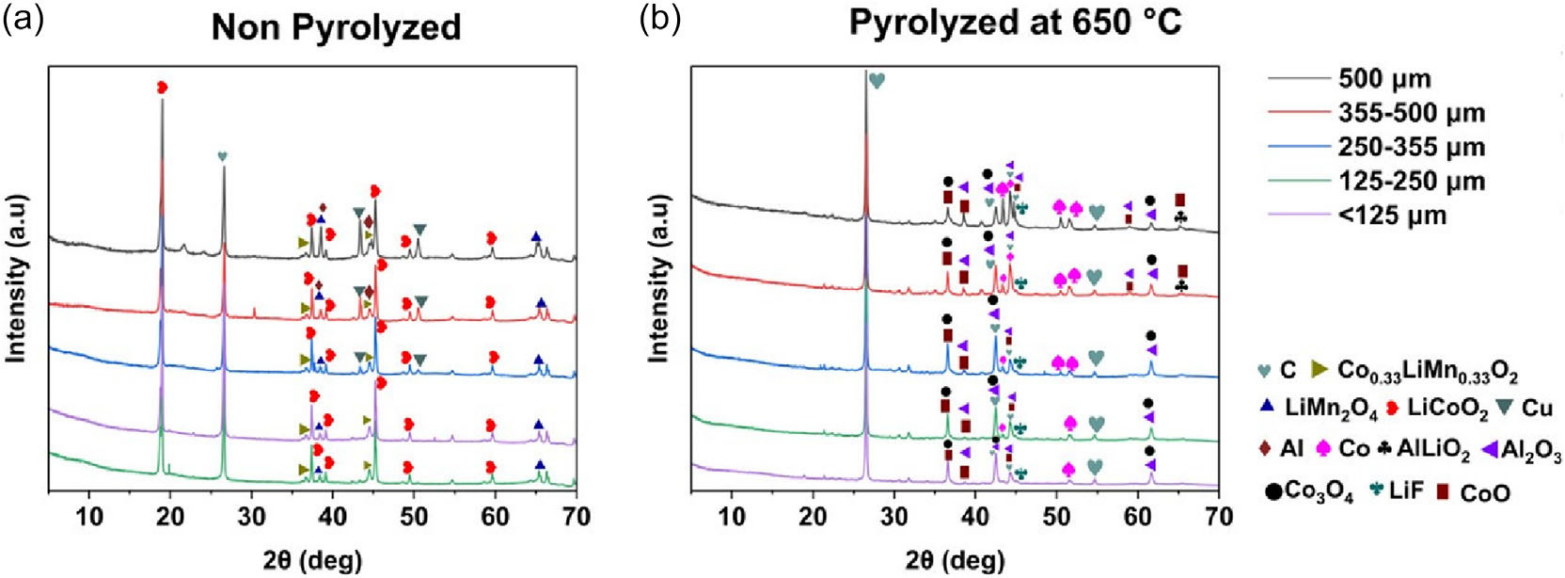
XRD patterns of BM (a) nonpyrolyzed and (b) pyrolyzed at 650°C for 30 min.

SEM images of selected regions of the >500 μm size fraction in polished cross‐sections were also analyzed and representative micrographs are shown in Figure 7. EDS analysis results are collected in Figures S5, S6, and Table S2. The images in Figure 7 show regions where cathode particles remain attached to Al foil current collectors. Before pyrolysis (Figure 7a), the foil was composed of Al in its metallic form. After pyrolysis at 600°C (Figure 7b), darker grey areas appear on the foil which, according to EDS analysis, represent areas of aluminum oxide. Simultaneously, the brighter hue at the cathode particle surfaces showcases a reduction into lower oxidation states or even their metallic forms. After pyrolysis at 650°C (Figure 7c), it was found that most of the Al foil was oxidized while the areas with lower oxidation states and pure metallic forms within the cathode particles became dominant, indicating aluminothermic reduction reactions taking place during the pyrolysis process at high temperatures. The disappearance of Al peaks after pyrolysis in XRD results presented in Figure 8b confirms such phenomenon. Figure 7c also shows a degradation of the physical integrity of Al, evidenced by the appearance of mechanical fractures. It is possible that the products of aluminum oxidation are comparatively fragile or generated in the form of fine powder. As the temperature is not high enough to cause sintering of this powder, some of it can be easily swept away from its original location.

In the study of Wang et al. [17], metallic Al current collector foils were shown to be efficient reductants of LiCoO_2_ cathode material in the temperature range hereby studied. The first reaction between LiCoO_2_ and Al can produce either Co_3_O_4_ or CoO, the latter being thermodynamically more favorable. In fact, according to Ellingham diagrams presented in the aforementioned work, the reduction of LiCoO_2_ with metallic Al takes place at lower temperatures than reduction with carbon. A potential side reaction is the formation of LiAlO_2_ from Li_2_O and Al_2_O_3_, although this could not be confirmed in the present work.

Figure 8 shows the XRD patterns of the pyrolysis products to further understand the structural and phase transformation observed in SEM‐EDS analysis. As seen in Figure 8a, the initial XRD pattern exhibits peaks corresponding to LiCoO_2_ and Al. However, at 650°C, these characteristic peaks are lost, while those corresponding to Al_2_O_3_, LiAlO_2_, Co_3_O_4_, CoO, and Co appeared, clearly indicating phase transformations resulting from carbothermic and aluminothermic reduction. This is well in accordance with the observations of SEM‐EDS.

Previously, Vanderbruggen et al. [57] studied pyrolysis of batteries in the same temperature range used in the current study. They observed partial melting of Al during the pyrolysis, indicating local temperatures higher than the melting point of Al (660°C) even though the maximum pyrolysis temperature was 650°C. This was attributed to unexpected thermal runaway during the process, but it is worth noting that they pyrolyzed entire battery cells (after discharging) instead of mechanically separated BM. Consequently, such study suggested that it may be beneficial to pyrolyze BM instead of entire batteries to avoid melting of Al and to increase the surface area of exposed cathode particles to enhance binder decomposition. During the current work, no evidence of Al melting was observed, which suggests that the violent release of thermal energy was avoided.

It is important to note that, while pyrolysis is frequently suggested as a pretreatment process for battery recycling, these results demonstrate that pyrolysis is not an inert process, as it induces chemical and structural transformations in electrode materials. In particular, the presence of carbonaceous species promotes carbothermic reduction of cathode active materials under the investigated conditions. For instance, several studies have treated BM via pyrolysis prior to flotation, assuming that this method was suitable for removal of binders and the subsequent liberation of cathode particles [57, 58]. Evidently, the scientific community has largely overlooked the reactivity between battery materials at pyrolysis temperatures, raising concerns about its suitability as pretreatment process for direct recycling methods.

### 3.4 Off‐Gas Composition

**FIGURE 9:**
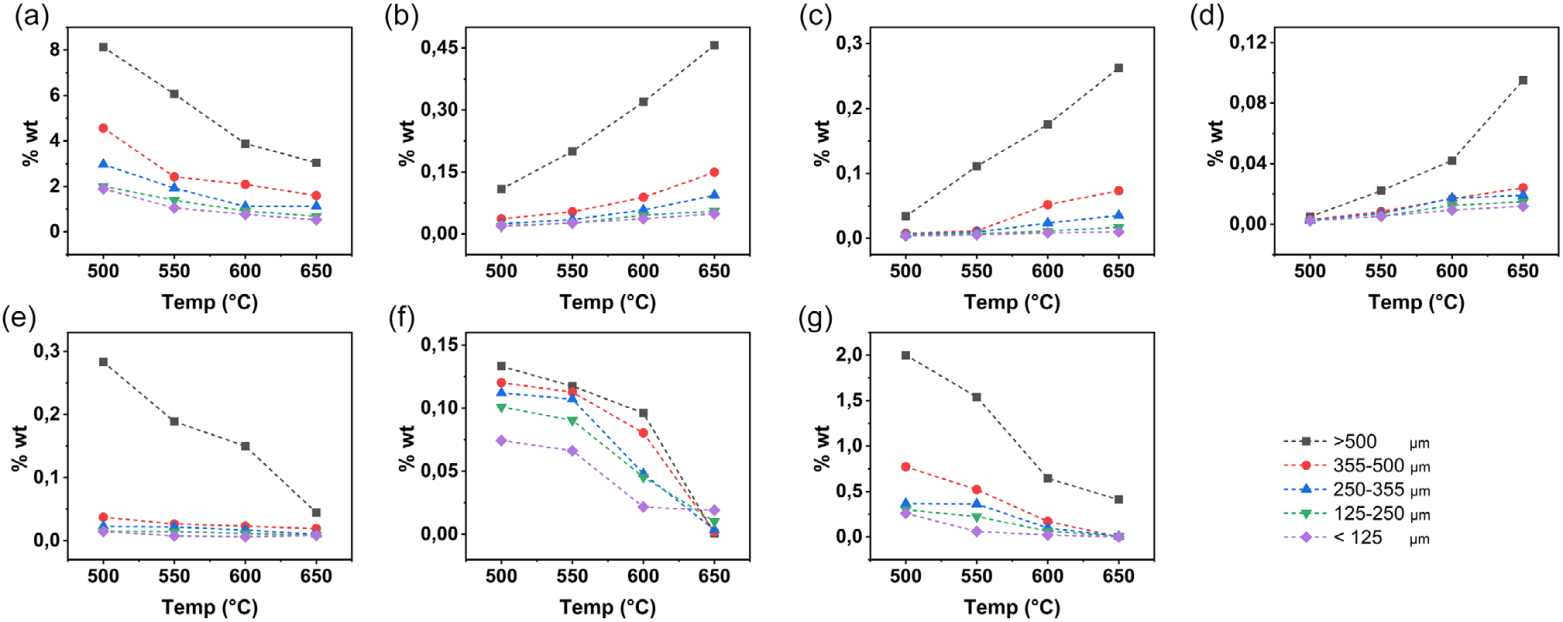
% weight analysis of the cumulative off‐gas composition at different temperatures over 30 min of pyrolysis time (a) CO_2_, (b) CO, (c) CH_4_, (d) C_2_H_4_, (e) C_2_H_6_, (f) C_3_H_8_, and (g) C_6_H_14_.

During pyrolysis experiments, the evolution of gases such as carbon oxides (CO_*x*_), methane (CH_4_), ethene (C_2_H_4_), ethane (C_2_H_6_), propane (C_3_H_8_), and hexane (C_6_H_14_) was identified. Figure 9 presents the cumulative concentrations of these species in the off‐gas streams produced during 30 min pyrolysis treatment. The species included in Figure 9 were those formed at the highest concentrations. It should be noted that other gases could have also formed, but they were not identified either due to their nonpolar nature, or their low concentration. For example, Hu et al. [59] reported the formation of up to 46 gases during pyrolysis of battery scrap at 400°C, the main species being consistent with those identified in the present work. Admittedly, a minor quantification error is present in Figure 9 since a fraction of some gaseous species might have condensed at the end of the sampling tube due to the comparatively lower temperature. It is, however, impractical to quantify the mass or composition of these condensed materials.

Regarding the source of the main gaseous species identified, the emission of CO_*x*_ can only be attributed to the carbothermic reduction of metal oxides and electrolyte thermal breakdown since pyrolysis was performed in an inert environment [36]. Furthermore, the generation of hydrocarbons is associated with volatilization of organic solvents, degradation of separators, electrolyte decomposition, and depolymerization of binder material [59, 60]. Notably, the formation of HF (g) was not detected under any experimental conditions, possibly due to its reaction with metal oxides and the formation of LiF identified in Figure 8 and previously reported by Huang et al. [33]. Figure 9 shows for the first time that the evolution of decomposition gases is sensitive to both size fraction and temperature. Among all gases analyzed, CO_2_ was the dominant species produced during all experiments. This is an expected byproduct of the carbothermic reduction of metal oxides with graphite or other forms of carbon present in the BM, producing CO_2_ and CO as indicated by Li et al. [36] and Gomez‐Moreno et al. [50]. Across all size fractions, the generation of CO_2_ decreased at higher temperatures, while the formation of CO was favored. This trend aligns well with the thermodynamic description reported by Lombardo et al. [61], where elevated temperatures decrease the Gibbs free energy (ΔG) favoring those reactions producing CO, in this case resulting from the reduction of LiCoO_2_, LiNi_0.5_Mn_0.3_Co_0.2_O_2_, and LiMn_2_O_4_. Additionally, the Boudouard reaction also contributes towards the formation of CO at elevated temperatures as demonstrated by Park et al. [56].

Similarly, there also appears to be a complementary relationship among the production of shorter hydrocarbon molecules such as CH_4_ and C_2_H_4_ compared to longer hydrocarbon chains, such as C_2_H_6_, C_3_H_8_, and C_8_H_14_. Invariably, as pyrolysis temperature increases from 500°C to 650°C, the generated amounts of methane (CH_4_) and ethene (C_2_H_4_) increased, while the emission of other hydrocarbons such as ethane (C_2_H_6_), propane (C_3_H_8_), and hexane (C_6_H_14_) was reduced. Such transformation of hydrocarbon gases can be reasonably explained by the thermal cracking of long chain hydrocarbons at elevated temperatures, promoting the formation of CH_4_ and C_2_H_4_ (Moldoveanu et al. [62]; Cassady et al. [63]; Nagaraja et al. [64]). Nevertheless, the quantification of these gases, most of which are toxic, and flammable will contribute towards process safety, risk assessment, and complying with environmental regulations.

### 3.5 Decomposition of PVDF Binder

**FIGURE 10:**
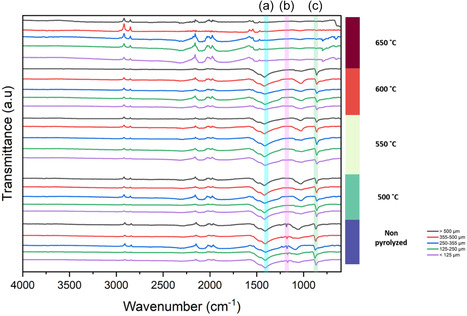
FTIR‐ATR spectra of size fractions pyrolyzed at different temperatures for 30 min: (a) functional group CH_2_, (b) and (c) functional groups CF_2_ for PVDF binder.

**FIGURE 11:**
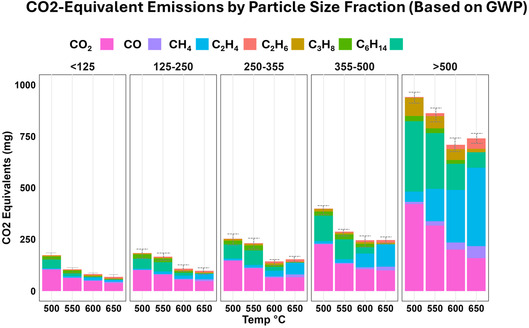
CO_2_ equivalents of pyrolyzing different BM size fractions at different temperatures for 30 min.

FTIR analyses were performed to study the behavior of PVDF binder during pyrolysis. Three signature peaks were considered representative of PVDF binder in battery waste: 870 cm^−1^ and 1180 cm^−1^ corresponding to functional group of CF_2_, and 1399 cm^−1^ ascribed to CH_2_ [65]. As indicated in Figure 10, all three peaks were identified in the nonpyrolyzed BM indicating the presence of PVDF binder. Irrespective of the size fraction, the peak at 1180 cm^−1^ disappears at low pyrolysis temperature of 500°C, whereas the remaining two peaks disappear only at pyrolysis temperature of 650°C. This indicates a two‐stage decomposition of PVDF binder. The partial decomposition at lower pyrolysis temperature, 400°C–500°C was also reported by Huang et al. [33]. As mentioned in the previous sections, since no HF gas formation was detected, fluorine species produced during binder decomposition likely reacted with LiCoO_2_, resulting in the formation of LiF and CoO, in line with the XRD analysis results shown in Figure 8. The formation of LiF is also thermodynamically favorable, since the Gibbs free energy Δ*G* ^0^ of LiF formation is lower as compared to HF formation under all experimental conditions of this work. These findings corroborate the conclusions by Huang et al. [33] that fluorine stays within the BM after pyrolysis in the form of metal fluoride. Our findings are also in agreement with the recent results of Wei et al. [66], who demonstrated that complete fluorine removal occurs only at ≥800°C and after 6 h of pyrolysis time. Some of the CoO shown in the XRD‐results (Figure 11) is most likely a product of aluminothermic/carbothermic reduction of the cathode material particles, as discussed in Section 3.3.

According to the FTIR results and supported by the XRD analyses, decomposition of PVDF binder occurs at 650°C (after 30 min of pyrolysis), along with the formation of LiF. It should be noted that decomposition and complete removal are not synonyms: according to SEM‐EDS results, binder residues are present after pyrolysis, even if their chemical structure was modified during pyrolysis.

Based on the results presented in Figures 10 and 11 and using an analogous PVDF degradation mechanism to the one proposed by Chen et al. [67], the following decomposition mechanism for PVDF binder during LIBs pyrolysis is proposed

(2) $${\text{(‐CH}}_{\text{2}}‐{\text{CF}}_{\text{2}}{\text{‐) + LiCoO}}_{\text{2}}\overset{\text{Δ}}{\to }\text{(‐CH = CF‐) + LiF}+\text{(1/3)}{\text{Co}}_{\text{3}}O_{\text{4}}{\text{+ (2/3)H}}_{\text{2}}O$$

(3) $${\left(‐\mathrm{CH}=\mathrm{CF}‐\right)}_{n}+H_{2}O+{\mathrm{Co}}_{3}O_{4}\overset{\text{Δ}}{\to }{\left(‐\mathrm{CH}=\mathrm{CF}‐\right)}_{n‐(q‐r‐s)}+q\mathrm{CO}+r{\mathrm{CO}}_{2}+sC_{x}H_{y}+3\mathrm{Co}$$

where the subscripts x and y represent an arbitrary number of carbon and hydrogen atoms in the hydrocarbon products. These products may include compounds such as CH_4_, C_2_H_4_, C_2_H_6_, C_3_H_8_, and C_6_H_14_ depending on the pyrolysis conditions as described in Section 3.4.

### 3.6 Evaluation of Environmental Impact of Pyrolysis

As part of the impact analysis of BM pyrolysis, it is necessary to determine whether the gas emissions pose threats to worker safety and the environment. Many of the gaseous species emitted during LIBs pyrolysis exhibit relatively high global warming potential (GWP). For example, the GWP (100‐year horizon, relative to CO_2_ = 1) for methane is 30, while it is 2 for CO, 7 for C_2_H_4_, 5.5 for C_2_H_6_, 3 for C_3_H_8_, and 5 for C_6_H_14_ [68, 69]. Figure 11 compiles the GWP in terms of CO_2_‐equivalent emissions resulting from pyrolysis of various size fractions at different treatment temperatures. The reported emissions are presented as mean values of three independent measurements (*n* = 3), with uncertainties calculated by combining the standard deviation and instrument error (±3% of the measured value) using a root‐sum‐of‐squares approach (Equation (1)). As seen, pyrolysis of larger size fractions under all experimental conditions resulted in higher GWP compared with the smaller size fractions. This is understandable since the larger size fractions contained a higher concentration of impurities. Consequently, advanced off‐gas handling processes may be needed during thermal treatment of these fractions. The total GWP starts decreasing as the temperature is increased, primarily caused by the lower amount of CO_2_ produced, as explained in section 3.4. This suggests that high temperatures may be advantageous from the overall GWP perspective. However, contrary to CO_2_, the impact of CH_4_ on GWP increased significantly at higher temperature, eventually offsetting the reduced impact of CO_2_. While lower CO_2_ emissions suggest a reduced climate impact, the increase in CH_4_, an extremely potent greenhouse gas requires careful consideration. This underscores that future work on the topic may need to incorporate additional metrics beyond GWP such as statistical entropy combined with life cycle assessment (LCA) [44], and exergy analysis [70, 71] to comprehensively compare the environmental impact of the emitted gases at various pyrolysis conditions. Nevertheless, the obtained information highlights a further operational challenge regarding the use of pyrolysis as pretreatment method for LIBs. Indeed, off‐gas treatment protocols are needed to comply with regulatory policies aimed at reducing and minimizing greenhouse gas emissions and addressing climate change.

In summary, the results hereby obtained indicate a multistage decomposition of binder and cathodic lattice. FTIR analysis demonstrated that partial decomposition of binder begins at 500°C and complete degradation occurred at 650°C after 30 min. Nevertheless, the effectiveness of high temperature for the removal of the binder comes along with the phase transformation of the cathode active material lattice. While cathode degradation was observed at 500°C, it becomes significantly more pronounced at higher temperatures (650°C) due to integrated reduction effects of carbothermic and aluminothermic reduction. This indicates that complete removal of binder is detrimental to cathode lattice structure preservation, which is key to direct recycling. Thus, if pyrolysis is to be considered as a pretreatment option, a partial decomposition of binder at lower temperatures might be preferred. A temperature range between 550°C–600°C seems to offer the best compromise between binder modification, cathode preservation, and process sustainability. If energy costs are also a significant factor, the partial binder degradation at 550°C may suffice to alter the BM properties, such as the hydrophobic nature of cathode particles, in preparation for froth flotation.

## 4 Conclusions

This study provides a first in‐depth, critical evaluation of pyrolysis as a pretreatment method for the removal of volatile components, polymers, and binders from battery active materials. To that aim, the impact of pyrolysis temperature and time on various size fractions of BM was studied, supported by a detailed characterization of products and gas emissions. The findings demonstrate that during pyrolysis, the cathode particles undergo carbothermic or aluminothermic reduction by reacting with battery components such as binder, Al, and graphite. The presence of PVDF binder likely contributed towards the reduction of lithium metal oxide, resulting in the formation of LiF and CoO. The results suggest that the degradation kinetics are faster under higher pyrolysis temperatures and most of the weight loss occurs during the initial 30 min of pyrolysis time, after which the weight loss is marginal. The largest size fractions presented the highest mass losses, likely due to the higher concentrations of electrolyte, separator, and plastics compared to smaller fractions. While almost complete decomposition and removal of binder was achieved at 650°C and 30 min, as indicated by FTIR, the analysis with SEM indicated a localized presence of residue binder. Thermal degradation of these components together with carbothermic reduction of metal oxides resulted in the emission of CO(g), CO_2_ (g) and various hydrocarbon gases. At higher temperature, larger chains of hydrocarbons undergo thermal cracking, favoring the production of shorter ones. SEM‐EDS and XRD analysis indicated that the cathode particle chemistry is altered to lower or higher degree due to reduction under all pyrolysis conditions hereby studied. Consequently, pyrolysis under such conditions should be more appropriately considered, at least partially, as a carbothermic or aluminothermic reduction process rather than an inert thermal treatment. Therefore, pyrolysis might not be suitable when the preservation of the chemical and structural integrity of materials is needed, for instance in the so‐called direct recycling routes.

The impact of pyrolysis in terms of GWP of the emitted gases was also reported. Notably, higher temperature conditions reduce the overall CO_2_ equivalents, with the largest size fraction (i.e., >500 μm) generating five times more CO_2_ equivalent emissions compared to smallest size fraction (i.e., <125 μm). Therefore, alternative pretreatment routes should be opted for larger size fractions to reduce environmental impact. The information obtained in terms of gas emissions and their quantification contributes to identifying the underlying chemical reactions and degradation pathways. These insights can subsequently contribute to improved industrial operations, thereby achieving better efficiencies, and optimizing pyrolysis temperature and duration, consequently lowering energy consumption. The research work also holds promise for LCA studies of spent LIBs. Future work will be conducted regarding the catalytic effect during pyrolysis due to variable composition of size fractions and on downstream processing stages, such as flotation for separating the graphite and cathode active materials from each other. These outcomes can contribute significantly to our understanding of the pyrolysis process within waste processing of spent LIBs.

## Author Contributions

**Mohazzam Saeed**: conceptualization, methodology, investigation, writing – original draft. **Lassi Klemettinen**: investigation, writing – original draft. **Anna Klemettinen**: investigation, writing – original draft. **Rodrigo** **Serna‐Guerrero**: supervision, funding acquisition, writing – review and editing.

## Funding

This study was supported by Business Finland (1754/31/2024).

## Conflicts of Interest

The authors declare no conflicts of interest.

## Supporting information

Supplementary Material

## Data Availability

The data that support the findings of this study are available on request from the corresponding author. The data are not publicly available due to privacy or ethical restrictions.
