# Supplementary material for: A Critical Appraisal of Pyrolysis Pretreatment in Lithium‐Ion Battery Recycling: From High‐Temperature Material Transformations to Environmental Impact
Source: ChemSusChem. 2026 Jun 7;19(11):e70789. doi: 10.1002/cssc.70789 (PMC13242870; doi:10.1002/cssc.70789)
Supplement: Supplementary file 1 — Supplementary Material [file CSSC-19-e70789-s001.pdf]

Supplementary material for

# A critical appraisal of pyrolysis pretreatment in lithium-ion battery recycling: from high-temperature material transformations to environmental impact

Mohazzam Saeed[1], Lassi Klemettinen[1], Anna Klemettinen[1] & Rodrigo Serna-Guerrero[1]

[1]Department of Chemical and Metallurgical Engineering, School of Chemical Engineering, Aalto University, P.O. Box 16200, Espoo, Aalto 0076, Finland

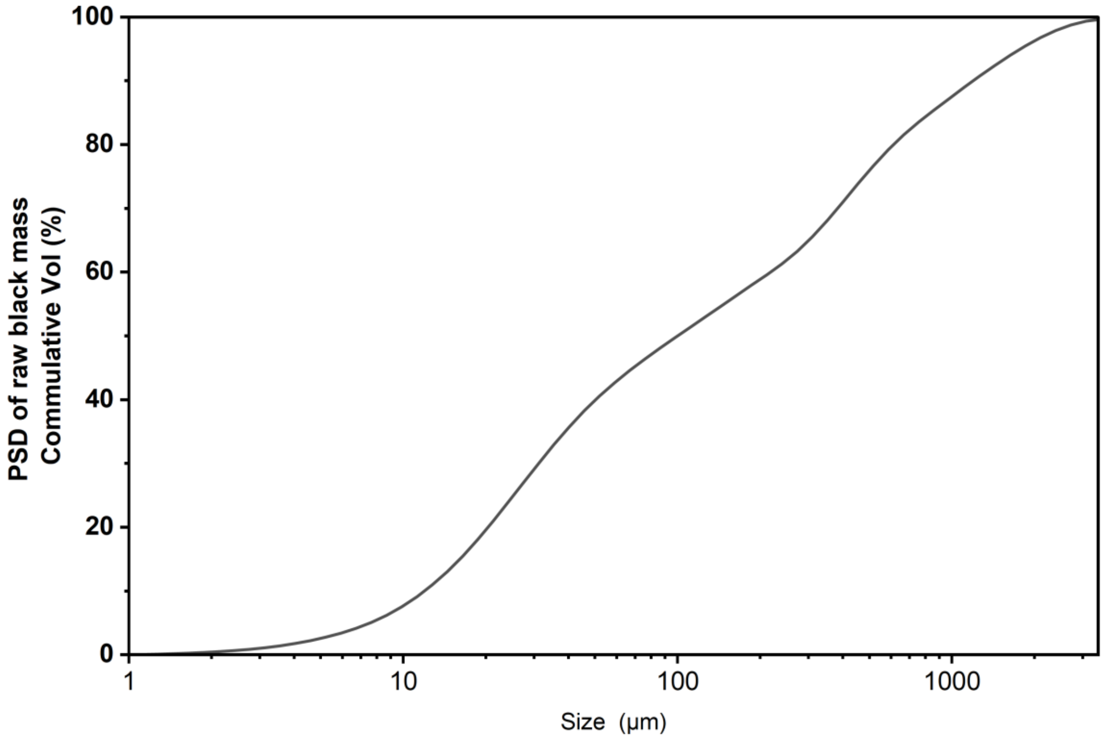


Figure S1. Cumulative particle size distribution (PSD) curve of the raw material.

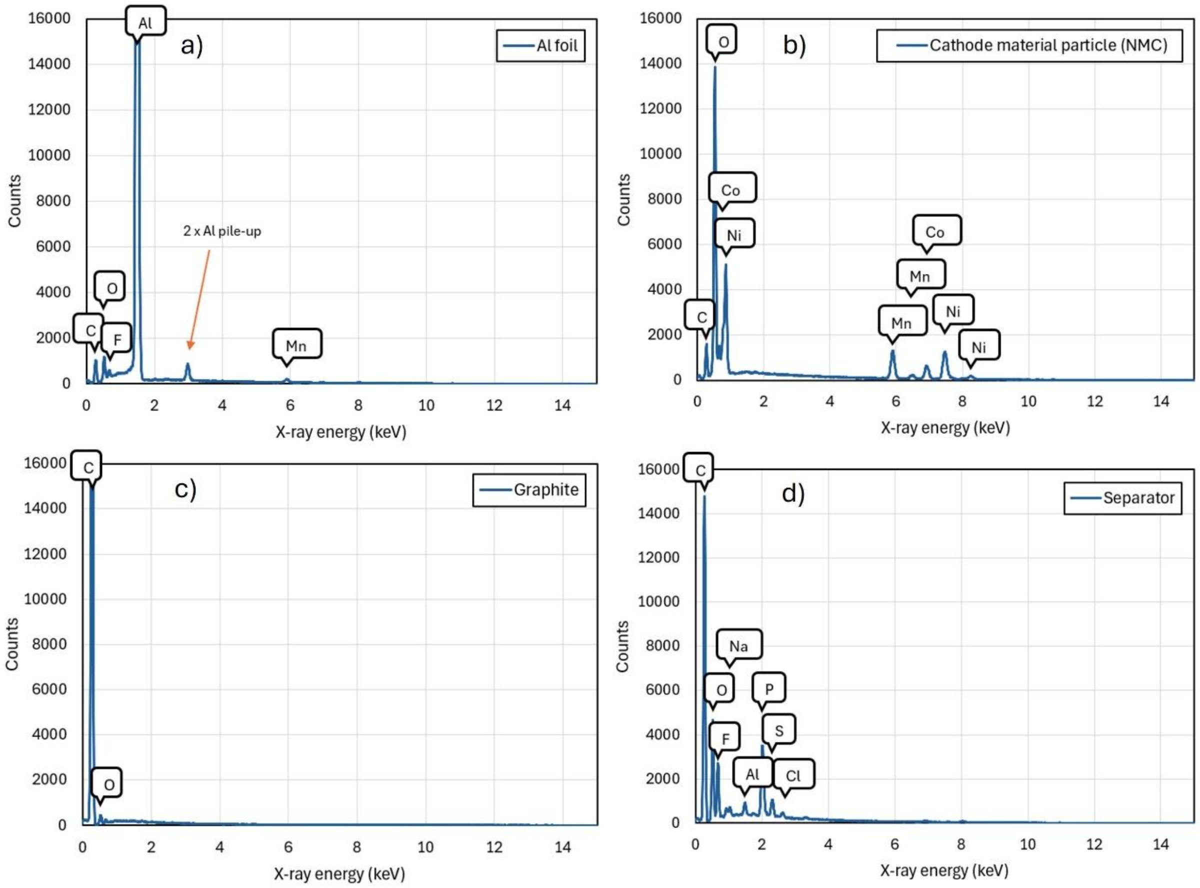


Figure S2. Examples of EDS spectra from different particles represented in Figure 4 of the manuscript. a) Al foil, b) cathode material particle (in this case NMC), c) graphite flake, d) separator. The results are not quantitative, as the sample surface was not flat.

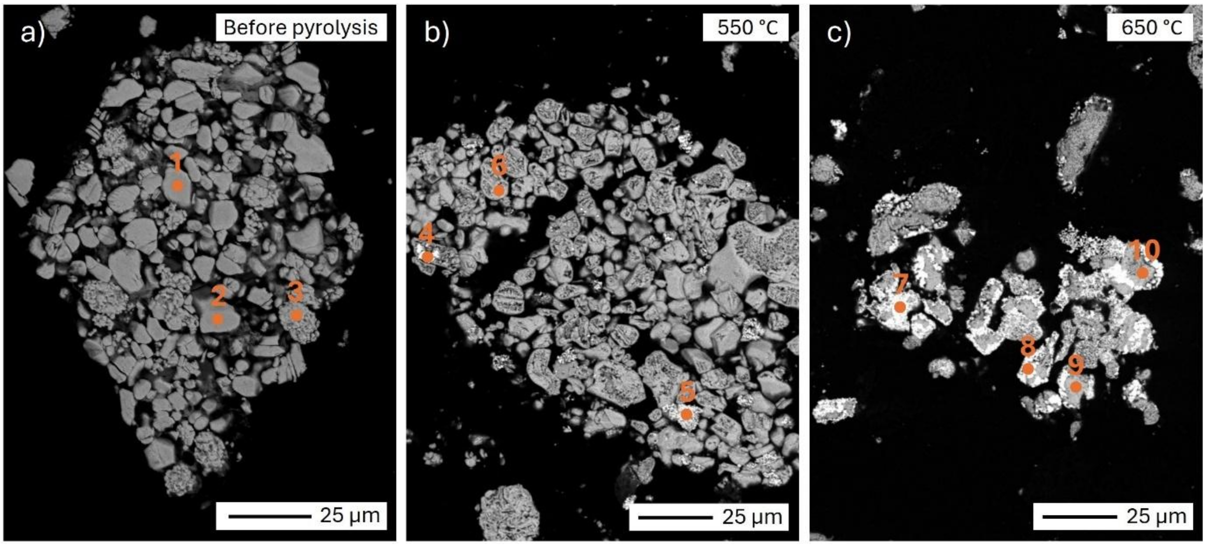


Figure S3. SEM-BSE micrographs of the same particles shown in Figure 6 of the manuscript. The orange dots indicate the locations where EDS analyses were obtained.

Table S1. Quantitative EDS analysis results (at%) from the locations indicated in Figure S3. Spectrums 1-2 are $LiCoO_2$ (Li cannot be analyzed using EDS); 3 is NMC532; 4, 5, 7 and 8 are areas where $LiCoO_2$ has been reduced to (almost) metallic Co; 6, 9 and 10 are areas where $CoO_2$ from $LiCoO_2$ has been reduced to CoO.

| | | | | | at% | | | | | | |
|---|---|---|---|---|---|---|---|---|---|---|---|
| **Label** | **O** | **F** | **Mg** | **Al** | **Si** | **P** | **Ca** | **Mn** | **Co** | **Ni** | **Zn** |
| **1** | 63.3 | 0.0 | 0.1 | 0.1 | 0.0 | 0.0 | 0.0 | 0.3 | 35.8 | 0.4 | 0.1 |
| **2** | 63.2 | 0.0 | 0.0 | 0.0 | 0.0 | 0.0 | 0.0 | 0.2 | 36.0 | 0.4 | 0.1 |
| **3** | 50.9 | 0.5 | 0.0 | 0.3 | 0.1 | 0.0 | 0.0 | 12.6 | 10.9 | 24.6 | 0.0 |
| **4** | 4.1 | 2.1 | 0.0 | 0.1 | 0.1 | 0.0 | 0.0 | 0.5 | 93.1 | 0.0 | 0.0 |
| **5** | 13.9 | 1.5 | 0.1 | 0.1 | 0.2 | 0.0 | 0.6 | 0.3 | 83.2 | 0.2 | 0.0 |
| **6** | 42.9 | 3.2 | 0.0 | 0.2 | 0.0 | 0.0 | 0.1 | 0.2 | 53.1 | 0.3 | 0.0 |
| **7** | 8.7 | 1.9 | 0.1 | 0.2 | 0.0 | 0.1 | 1.1 | 0.8 | 86.8 | 0.3 | 0.0 |
| **8** | 2.9 | 2.3 | 0.1 | 0.2 | 0.1 | 0.0 | 0.3 | 0.5 | 93.3 | 0.4 | 0.0 |
| **9** | 46.5 | 3.6 | 0.4 | 0.2 | 0.1 | 0.0 | 0.7 | 1.9 | 45.9 | 0.4 | 0.3 |
| **10** | 43.5 | 3.6 | 0.5 | 0.3 | 0.1 | 0.0 | 0.0 | 1.4 | 50.0 | 0.3 | 0.3 |

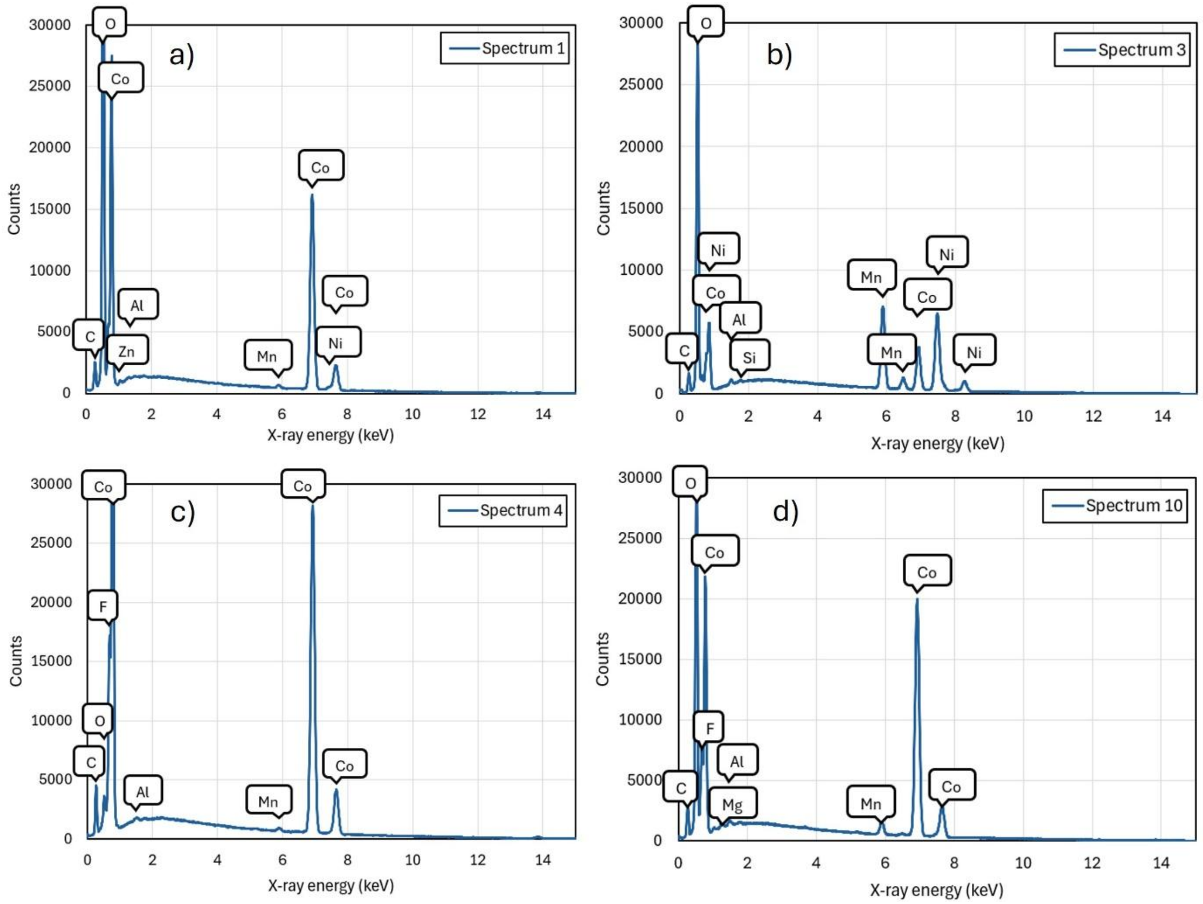


Figure S4. Examples of EDS spectra from locations marked in Figure S3. The corresponding, quantitative elemental compositions are given in Table S1. a) $LiCoO_2$, b) NMC532, c) metallic Co, d) CoO. In Figures c) and d), nothing can be said about the oxidation state of Li, as it cannot be analyzed using EDS. The y-axis scale has been limited to 30 000 counts in order to show the small peaks more clearly.

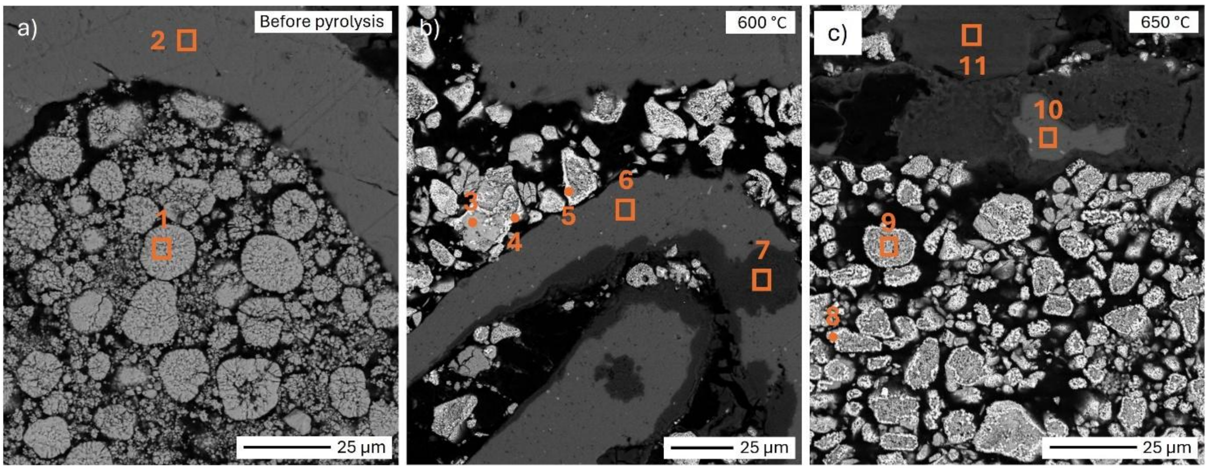


Figure S5. SEM-BSE micrographs of the same particles shown in Figure 7 of the manuscript (with different magnification). The orange dots indicate the locations where EDS analyses were obtained.

Table S2. Quantitative EDS analysis results (at%) from the locations indicated in Figure S5. Spectrum 1 is NMC532, spectrums 2, 6 and 10 are metallic Al, spectrum 3 is an area where $CoO_2$ from $LiCoO_2$ has been reduced to CoO or even close to $Co_2O$ (spectrum 9), spectrums 4, 5 and 8 are (almost) metallic Co, and spectrums 7 and 11 are showing oxidized Al foil.

| | at% | | | | | | | | | | |
|---|---|---|---|---|---|---|---|---|---|---|---|
| **Label** | **O** | **F** | **Mg** | **Al** | **Si** | **P** | **Ca** | **Mn** | **Co** | **Ni** | **Zn** |
| **1** | 61.3 | 2.7 | 0.0 | 0.0 | 0.1 | 0.0 | 0.0 | 10.5 | 7.1 | 18.2 | 0.0 |
| **2** | 3.4 | 0.1 | 0.0 | 95.8 | 0.0 | 0.1 | 0.0 | 0.2 | 0.1 | 0.3 | 0.0 |
| **3** | 46.6 | 3.5 | 0.7 | 0.2 | 0.1 | 0.0 | 0.6 | 0.0 | 47.8 | 0.3 | 0.1 |
| **4** | 6.9 | 2.1 | 0.2 | 0.9 | 0.2 | 0.1 | 0.1 | 0.1 | 89.5 | 0.0 | 0.0 |
| **5** | 15.0 | 1.9 | 0.7 | 0.4 | 0.2 | 0.0 | 0.1 | 0.1 | 81.7 | 0.0 | 0.0 |
| **6** | 3.3 | 0.0 | 0.0 | 96.3 | 0.1 | 0.1 | 0.0 | 0.0 | 0.3 | 0.0 | 0.0 |
| **7** | 57.7 | 9.0 | 0.0 | 30.4 | 0.7 | 1.9 | 0.1 | 0.0 | 0.1 | 0.0 | 0.1 |
| **8** | 8.2 | 1.4 | 0.0 | 0.1 | 0.1 | 0.0 | 0.0 | 0.0 | 90.2 | 0.0 | 0.1 |
| **9** | 35.9 | 3.3 | 0.3 | 0.2 | 0.0 | 0.1 | 0.0 | 0.1 | 59.8 | 0.1 | 0.1 |
| **10** | 2.8 | 0.2 | 0.0 | 96.2 | 0.6 | 0.1 | 0.0 | 0.0 | 0.3 | 0.0 | 0.0 |
| **11** | 64.3 | 0.5 | 0.1 | 31.9 | 0.6 | 1.1 | 0.4 | 0.1 | 1.1 | 0.0 | 0.1 |

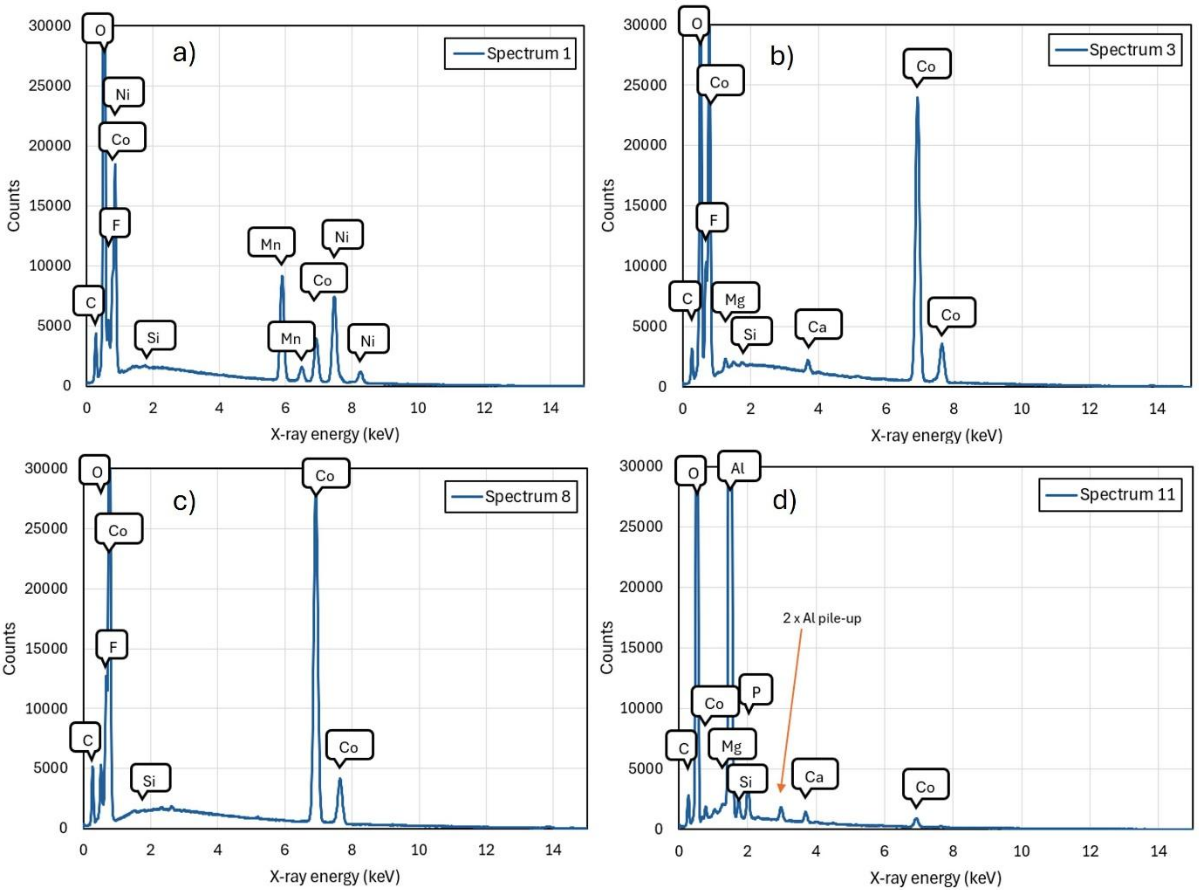


Figure S6. Examples of EDS spectra from locations marked in Figure S5. The corresponding, quantitative elemental compositions are given in Table S2. a) NMC532, b) CoO, c) metallic Co, d) oxidized Al foil. In Figures b) and c), nothing can be said about the oxidation state of Li, as it cannot be analyzed using EDS. The y-axis scale has been limited to 30 000 counts in order to show the small peaks more clearly.
